# Elevated Intracranial Pressure as a Cause of Sick Sinus Syndrome

**DOI:** 10.1155/2018/5015840

**Published:** 2018-06-03

**Authors:** Samer Makhaly, Parvaneh Fallah, Nadia Giannetti

**Affiliations:** ^1^Research Institute of McGill University Health Center, McGill University, Montreal, QC, Canada; ^2^Department of Cardiology, McGill University Health Center, McGill University, Montreal, QC, Canada

## Abstract

Sick sinus syndrome (SSS) has multiple causes both familial and acquired. The most common cause is usually idiopathic. In the past literature, elevated intracranial pressure (ICP) has not been reported to be a cause of SSS. We present a case of a 55-year-old male that developed SSS after surgical resection of a brain tumor. We have investigated the causal relationship between increased ICP and SSS. We have concluded that elevated ICP creates a sympathovagal imbalance leading to SSS.

## 1. Introduction

Sick sinus syndrome is the collection of clinical conditions that result from an abnormality of the sinoatrial nodal automaticity. Although it is usually idiopathic, a number of intrinsic and extrinsic causes have been described in the literature [1]. We report a case with a rare cause of SSS and the underlying pathophysiology that led to this presentation.

## 2. Case Report

A 55-year-old male presented to the emergency department 3 weeks after right frontal nodule resection of his multifocal glioblastoma multiforme. The tumor was also in the corpus callosum and the right pontine area. The patient was known to have high ICP (confirmed by magnetic resonance imaging) due to the mass effect of the tumor for which a debulking surgery was done. Recovery from surgery went well, and the patient had a residual left-sided weakness and was able to ambulate by using a walker at discharge. At presentation to the emergency department, the patient had 2 episodes of unprovoked syncope with nausea, somnolence, and worsening of his left-sided weakness. The patient's past medical history was insignificant except for controlled hypertension and epilepsy. The patient's home medications were dexamethasone, levetiracetam, hydromorphone, perindopril, amlodipine, and pantoprazole. While in the emergency room, the patient was noted to have a temporary pause on the cardiac monitor which was symptomatic. The patient's vital signs on admission were a Glasgow Coma Scale of 15, blood pressure of 132/86, heart rate of 61, and respiratory rate of 18 (regular), afebrile, and an oxygen saturation at 94% on 2 L/min of oxygen. When the oxygen was removed, the patient's respiratory rate decreased to 12, oxygen saturation dropped to 89%, and the patient became nonalert. An EKG was done which showed multiple intermittent sinus pauses of durations of 4–6 seconds. The blood work and urine analysis were all within the normal range. A diagnosis of SSS was made, and the patient was admitted to the CCU for pacemaker implantation. While admitted to the CCU, his EKG showed intermittent sinus pauses with the longest having a duration of around 3.6 seconds. A pacemaker was inserted. A 24-hour monitoring post procedure was uneventful, and the patient's telemetry showed a normal sinus rhythm in the 80s to 90s with no paced beats. The patient was then discharged home to be followed up at the pacemaker clinic. During the follow-up, the patient's hemiparesis did not recover and was still using the walker to ambulate. A computed tomography (CT) scan done later showed rapid progression of all lesions, except the one in the pons, with vasogenic edema. There was a significant mass effect with midline shift and uncal herniation.

## 3. Discussion

Sick sinus syndrome can be caused by genetic or acquired etiologies, but the most common cause is idiopathic. SSS was found to be associated with aging (mean age of 68 years old) due to age-related degenerative fibrosis of the SA node [2]. This is a less likely cause of SSS in this patient due to his relatively young age. Another possible cause of SSS that is related to this case is temporal partial seizure which has been reported to induce sinus arrest [3]. However, the patient was on levetiracetam and did not show the usual aura symptoms associated with a temporal partial seizure. Thus, this was excluded as a cause. To the best of our knowledge, none of the medications mentioned above has been reported to cause SSS. With the absence of any reported causes, SSS in this case is most likely to be caused by an imbalance of the autonomic tone to the SA node [4]. This, in theory, could be subdivided into either hypoactivity of sympathetic nerve outflow or hyperactivity of parasympathetic nerve outflow. The sympathovagal imbalance can be due to the local pressure effect of the pontine lesion or due to an increase in the ICP from the multifocal tumor.

The autonomic nervous system has a major role in the pathogenesis of some of the extrinsic causes of SSS [5]. Vagally triggered sinus bradycardia secondary to increased ICP has been reported as early as 1901 by a well-known phenomenon known as “Cushing's reflex.” It is a response by the autonomic nervous system to ensure adequate cerebral perfusion despite increased ICP [6]. Elevated ICP, as a result of subarachnoid hemorrhage, has also been reported to affect the function of the autonomic nervous system. Kawahara et al. stated that the high ICP triggered an increased vagal discharge to the heart leading to sinus cycle abnormalities [7]. A similar physiologic process was used to explain the occurrence of sinus arrest during rapid eye movement sleep [8].

On the other hand, the sympathetic nervous system responds variably to increased ICP depending on the onset, duration, and severity of the intracranial hypertension. An acute elevation of the ICP leads to an initial response of increased heart rate, cardiac output, and blood pressure due to neurohumoral activation. With the continued increase in ICP, a triad of bradycardia, hypertension, and irregular breathing commences due to both sympathetic and vagal stimulations. Further elevation in the ICP to the degree of spinal ischemia leads to cardiovascular collapse [9–11]. These responses were observed with an ICP that was raised acutely and surpassing the mean arterial blood pressure. On a different note, Matsuura et al. concluded that with low to moderate ICP elevation, both arms of the autonomic nervous system are stimulated, where the parasympathetic stimulation is more profound [10, 12].

Brainstem lesions are known to cause cardiovascular autonomic dysfunction. A number of case studies have reported that compression of the vasomotor area occurs mainly due to lesions that lie within or close to the medulla oblongata. Both elevated ICP and brainstem lesions cause autonomic dysregulation via the same mechanism: brainstem compression [13–15]. A clinical picture of depressed global consciousness and respiratory depression in the context of a history of intracranial hypertension unresponsive to treatment indicates that the most likely cause of SSS in this case is elevated ICP.

It is clearly evident that the sympathetic and parasympathetic nervous systems influence the cardiovascular response to elevated ICP. Our patient did not present with a hyperdynamic state, and Cushing's response and papilledema were absent. This implies that a gradual low to moderate elevation in the ICP was present. Based on our review of the literature, we have concluded that SSS in our case was due to spiked parasympathetic predominance primarily due to increased vagal discharge.

ICP as a cause of SSS has been reported once in the literature by Takayama et al. [16]. The reported case had many factors that were more likely to cause SSS than the increased ICP. This included old age (84 years old) and ischemic heart disease [17]. In addition, SSS occurred just after the administration of vagomimetic anesthetic drugs, fentanyl and vecuronium, which was reported to cause SSS and is the most plausible cause in this scenario [18]. On the other hand, our patient was unique as there were no other confounding causes that might have precipitated SSS.

Our patient demonstrates an unusual cause of SSS. Recognizing that increased ICP can cause SSS is important so that a high degree of suspicion is maintained and a closer follow-up of patients at risk can be done. In addition, patients at risk of ICP elevation should avoid certain medications that can cause SSS to manage their other comorbidities.

We also recommend perioperative cardiac work up for elderly patients at risk of having elevated ICP, even in the absence of any cardiac risk factors or symptoms. This would include a 12-lead ECG to rule out any ECG abnormalities indicative of a silent SSS. Reports have recommended that in cases of silent SSS, patients would undergo pacemaker implantation before general anesthesia to protect them from any hemodynamic instability induced by the anesthetic drugs [19, 20]. If not, then vagomimetic anesthetic drugs must be used with caution to avoid hypervagotonic activity to the SA node.
